# Prospects for *Trifolium* Improvement Through Germplasm Characterisation and Pre-breeding in New Zealand and Beyond

**DOI:** 10.3389/fpls.2021.653191

**Published:** 2021-06-16

**Authors:** Lucy M. Egan, Rainer W. Hofmann, Kioumars Ghamkhar, Valerio Hoyos-Villegas

**Affiliations:** ^1^CSIRO Agriculture and Food, Narrabri, NSW, Australia; ^2^Faculty of Agriculture and Life Sciences, Lincoln University, Lincoln, New Zealand; ^3^AgResearch Grasslands Research Centre, Palmerston North, New Zealand; ^4^Department of Plant Science, Faculty of Agricultural and Environmental Sciences, McGill University, Montreal, QC, Canada

**Keywords:** *Trifolium*, pre-breeding, germplasm, genebank, germplasm characterisation, core collections

## Abstract

*Trifolium* is the most used pastoral legume genus in temperate grassland systems, and a common feature in meadows and open space areas in cities and parks. Breeding of *Trifolium* spp. for pastoral production has been going on for over a century. However, the breeding targets have changed over the decades in response to different environmental and production pressures. Relatively small gains have been made in *Trifolium* breeding progress. *Trifolium* breeding programmes aim to maintain a broad genetic base to maximise variation. New Zealand is a global hub in *Trifolium* breeding, utilising exotic germplasm imported by the Margot Forde Germplasm Centre. This article describes the history of *Trifolium* breeding in New Zealand as well as the role and past successes of utilising genebanks in forage breeding. The impact of germplasm characterisation and evaluation in breeding programmes is also discussed. The history and challenges of *Trifolium* breeding and its effect on genetic gain can be used to inform future pre-breeding decisions in this genus, as well as being a model for other forage legumes.

## Introduction

The *Trifolium* species are among the most important and valuable forage legumes in the world (Russel and Webb, 1976). The genus *Trifolium* includes more than 250 species with a handful of species having economic importance as forage (Zohary and Heller, 1984) and others having potential for use as future forages. The most widely used species within the genus are white clover (*Trifolium repens*) and red clover (*Trifolium pratense*). White clover is the most widely used pastoral legume in temperate zones of the world (Caradus et al., 1996b). It is grown extensively throughout pastoral systems in Europe, western Asia, North America, South America, Australia, and New Zealand. It is of value to agricultural pastures due to the high nutritional value and quality, persistence, wide climatic range of growth, high seed production and ability to fix atmospheric nitrogen (Williams, 1987). While white clover can be grown in a wide range of climates, it is mostly grown in mild to cold temperate climates (Frame and Newbould, 1986).

White clover is an allotetraploid (2*n* = 4*x* = 32) perennial legume and exhibits disomic inheritance. The genome size is compact (1C = 1,093 Mb) (Bennett and Leitch, 2011). White clover is a recent allopolyploid that arose 13,000–130,000 years ago through the hybridisation of two diploid ancestors; *Trifolium occidentale* and *Trifolium pallescens* (Ellison et al., 2006; Williams et al., 2012; Griffiths et al., 2019). The mating system is highly outcrossing with a gametophytic self-incompatibility system, which develops heterozygous populations (Abberton, 2007; Williams et al., 2012).

Red clover is found natively in Europe, western Asia and north-western Africa. It is grown widely as a fodder crop that is used for silage and hay. Red clover is a diploid (2*n* = 2*x* = 14) perennial legume with a genome size of ∼420 Mb (Ištvánek et al., 2014; De Vega et al., 2015). Like white clover, red clover is almost completely self-sterile and produces highly variable populations. Red clover cultivars can be diploid or tetraploid, with the tetraploid cultivars outperforming the diploid cultivars in some agronomic traits (Taylor, 2008).

**FIGURE 1:**
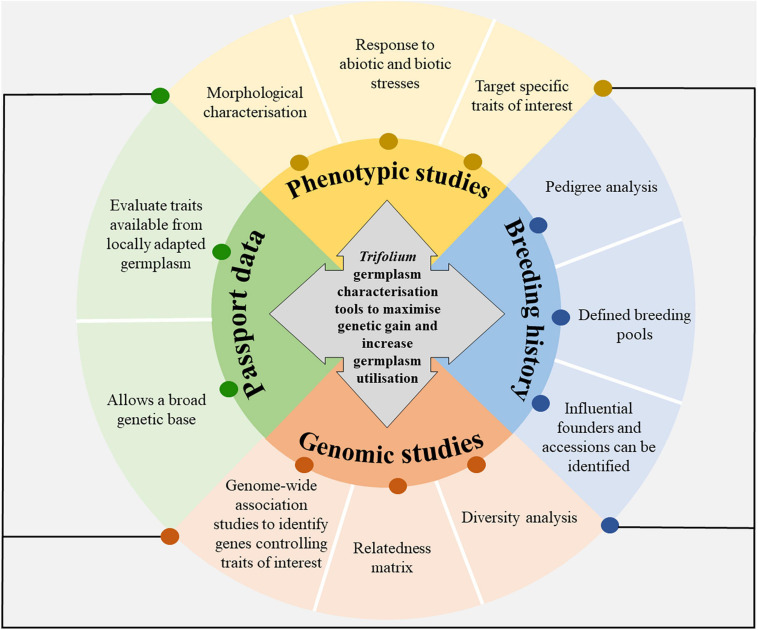
The deep characterisation of germplasm held in gene banks is targeted to increase germplasm utilisation and the flow of germplasm between institutions and countries. Core collections can be developed from the data produced from germplasm characterisation tools. Future management of germplasm banks will require increased germplasm exploration and characterisation.

The objective of this paper is to describe the role of genebanks in *Trifolium* breeding and what the characterisation of germplasm held in the genebanks means for plant breeding and pre-breeding. The paper will overview past breeding efforts, the importance of genebank and core collections, as well as suggesting avenues to characterise and utilise variation. Figure 1 shows the model framework for *Trifolium* germplasm characterisation and utilisation. Emphasis will be given to the application for New Zealand agriculture, but examples of research from other countries used in New Zealand as well as globally will be provided.

## Role of *Trifolium* Species in New Zealand Pastoral Systems

### White Clover

White clover is often described as the base legume for New Zealand’s pastoral sector. Annually, 1,000–1,200 tonnes of white clover seed is sold in New Zealand and 4,500 tonnes are exported around the world. New Zealand has the highest global export share of white clover seed (57.5%) (Rattray, 2005). The species is commonly used in mixture with grass for grazing. It is tolerant to a range of grazing systems, including dairy and beef cattle, sheep and deer, and is favoured due to its high feed value (Frame and Newbould, 1986). Traditionally, white clover has not been used in hay or silage. The brittleness of the leaves, lack of bulk production and problems with producing well-fermented silage has since been overcome by wilting, chopping and the use of acid additives (Williams, 1987).

The nitrogen-fixing capability of white clover is favourable in a sward as it reduces the need for synthetic fertilisers for the companion grasses. Crush (1987) estimated that white clover has the potential to fix 600–700 kg N/ha/year. However, varying abiotic and biotic conditions can lower nitrogen fixation rates (Lucas et al., 2010). White clover fixes approximately 1.57 million tonnes of nitrogen annually, contributing approximately $1.49 billion to the New Zealand economy (Caradus et al., 1996b).

Fluctuating herbage yields are the result of varying biotic and abiotic stresses such as pests and diseases, drought and differing grazing management, as well as introduced legislation restricting farming practices. Brown and Green (2003) identified one of the main constraints of white clover production as lack of persistence in drought. Warming global temperatures signifies the low performance of white clover under long-term drought (Macfarlane et al., 1990; Sheath et al., 1990; Brown and Green, 2003). The ideal growth temperature for white clover is 20–24°C, and when temperatures are not optimal, production decreases (Harris et al., 1985). The poor performance in drought has significant effects on its production. Studies show that white clover cultivars have fluctuating herbage yield every year in response to environmental stresses (Jahufer et al., 2002; Jahufer M. et al., 2013). This inconsistent performance has been attributed to poor survival through summer drought conditions (Robinson and Lazenby, 1976; Gillard et al., 1989).

### Red Clover

Red clover is a perennial clover with a large taproot and high feed value; 25–35% bypass protein (Hoffman and Broderick, 2001). Red clover is not as prevalent as white clover in New Zealand pastoral systems, although approximately 100–150 tonnes of red clover seed is sold in New Zealand annually (Rolston, 2003). Worldwide, it is primarily used as a fodder crop in the form of silage and hay. When used in a pastoral system, red clover is often mixed with white clover in pasture mixes (Kemp et al., 1999; Cassileth, 2010). Red clover does not persist or perform well in intensive grazing systems and is better suited for dry summer areas where less intensive grazing systems are applied (Kemp et al., 1999).

Red clover offers several advantages compared to white clover. These include: (i) faster establishment time and better performance in dry summer environments (Moot et al., 2000; Brown et al., 2005); (ii) more efficient water use in water-limited areas owing to the deep taproot (Taylor and Quesenberry, 1996a); (iii) higher tolerance to pasture pests (Gerard et al., 2017), and (iv) improved nitrogen partitioning when consumed by livestock (Sullivan and Hatfield, 2006; Van Ranst et al., 2011). However, the biggest inhibitor of red clover performance in pastoral systems is the lack of persistence (Ford and Barrett, 2011). The longevity of red clover is generally 2–3 years, and when sub-optimal pasture conditions are present, persistence continually decreases (Hyslop et al., 1999). Red clover persistence is reduced with frequent, hard grazing. Continuous grazing reduces the carbohydrates available in the taproot and increases the vulnerability of the plant to disease. Smith (1989) stated that increased persistence in red clover was correlated with an increased number of plants with more fibrous root systems. The survival and performance of the taproot is a vital aspect of the survival and performance of the plant as a whole (Smith et al., 1985; Brock et al., 2003).

Pests and diseases are most rampant during the establishment of red clover. However, the long-term toll of diseases is a critical issue for red clover. As the sward increases in cover, red clover is more competitive and less susceptible to infection in the sward (Frame, 2019). The predominant crown disease (*Selerotinia trifoliorum*) and root diseases (*Fusarium* spp. and *Rhizoctonia* spp.) are the major diseases of red clover. At the same time, weevils and nematodes are major pests (Hyslop et al., 1999). Often, wounding of the plants from grazing animals leaves the plant open and susceptible to infection and damage. However, usually, the late-flowering cultivars have higher persistence and lead to less damage from pests and diseases early in establishment. There is a positive correlation between ploidy levels and increased resistance to pests amongst red clover cultivars (Hay and Ryan, 1989).

A common animal health issue from grazing red and white clover is bloat. Bloat is often more common in cattle than sheep. The high levels of protein form general gas in the stomach of the animal and when the gas levels become more elevated than the animals’ ability to expulse the gas, bloat occurs (Majack et al., 2003). Pasture mixes containing grass and grazing management plans are utilised to reduce the risk of bloating (Frame and Newbould, 1986).

### Minor *Trifolium* Species

Many *Trifolium* species are underutilised, or their use is not yet considered economically viable in agricultural systems (Maxted and Bennett, 2001). The importance of the minor species within the *Trifolium* genus is, however, recognised for: (i) growth in adverse conditions; (ii) research to improve the major species; or (iii) hybridisation with major *Trifolium* species (Morris and Greene, 2001; Ellison et al., 2006; Williams, 2014; Egan et al., 2020).

*Trifolium subterraneum* and *Trifolium ambiguum* are two species that have become popular to use in drought-prone pastoral systems of Australasia and North America (Knight et al., 1982; Virgona and Dear, 1996; Reed et al., 2001; Nichols et al., 2013; Hayes et al., 2020). *Trifolium subterraneum*, known as “subterranean clover” or “sub clover,” is an annual forage legume species of clover native to the Mediterranean region and western Asia. Of all the annual clovers, subterranean clover has the highest contribution to livestock feed production (Kaur et al., 2017). Subterranean clover is a highly self-pollinating, diploid (2*n* = 16) species (Ghamkhar et al., 2012; Hirakawa et al., 2016; Kaur et al., 2017). *T. subterraneum* is a species that is highly utilised in Australia and to a lesser extent in New Zealand (Nichols et al., 2007; Lucas et al., 2015; Olykan et al., 2018). *T. subterraneum* is well characterised to perform in dry conditions as it buries its burrs in the soil, allowing seed development to happen underground (Suckling et al., 1983; Widdup and Pennell, 2000). *Trifolium ambiguum*, is commonly known as “Caucasian clover” or “Kura clover.” *T. ambiguum* can be diploid (2*n* = 16), tetraploid (2*n* = 32) or hexaploid (2*n* = 48) (Bryant, 1974; Taylor and Smith, 1997). Although the ploidy of the species affects some traits, such as flowering date and persistence, the yield is unaffected (Bryant, 1974; Dear and Zorin, 1985). *T. ambiguum* has a large root system and is favourable in agricultural systems that are exposed to drought conditions (Bryant, 1974; Maxted and Bennett, 2001). *T. ambiguum* has been used to hybridise with white clover (Ellison et al., 2006; Williams, 2014). The *T. repens* × *T. ambiguum* interspecific hybrids have been successful in producing progeny that have advantageous root characteristics (Abberton et al., 1998), and similar forage quality (Marshall et al., 2004) and nitrogen fixation capacity to white clover (Abberton et al., 2000).

*Trifolium arvense, Trifolium dubium, Trifolium hybridum*, and *Trifolium medium* are four *Trifolium* species that are used primarily for research to understand and improve other *Trifolium* species. *T. arvense*, commonly known as “rabbitfoot clover” or “hares-foot clover,” is an annual winter clover that is native to Europe and western Asia and grows well in sandy or non-irrigated land (Pritchard et al., 1988). The optimal environment in pastoral systems for *T. arvense* is short-lived, low fertility pastures (Taylor, 1985; White and Hodgson, 1999). *T. arvense* can survive for long periods under intense grazing but is often outcompeted by grasses under lighter grazing (Palmer, 1972). *T. dubium*, known as “suckling clover,” is an allotetraploid (2*n* = 4*x* = 30) clover that is native to Europe (Bulińska-Radomska, 2000). It arose from a cross of *T. campestre* and *T. micranthum* (Hedlund et al., 2003). *T. dubium* is often found in the low fertility hill country (Caradus and Mackay, 1989). *T. hybridum*, commonly known as “alsike clover,” is a clover that originates from Europe and has established throughout temperate regions of the world (Williams, 1951). *T. hybridum* is a highly self-sterile, short-lived perennial clover (Williams, 1951). It is adaptable to a wide range of conditions and has rapid establishment (Widdup and Ryan, 1994). Therefore, it is often used in the hill country of the South Island in New Zealand for pasture and hay or silage (Williams, 1951; Taylor, 1985). *T. medium*, commonly known as “zigzag clover,” is a native European perennial species with the ploidy of 2*n* = 10*x* = 80 (Merker, 1984; Isobe et al., 2002). It is similar in appearance to red clover but with narrower leaflets and no white leaf markings (Choo, 1988). *T. medium* is a long-lived perennial clover that prefers damp, acidic soils (Taylor, 1985). *T. medium* is known to be among the most persistent clover species (Choo, 1988). Although not common in commercial pastoral systems, *T. medium* has the potential for pasture and hay production (Taylor et al., 1984).

## History of *Trifolium* Breeding in New Zealand

Breeding for *Trifolium* species in New Zealand commenced in the early 1900s. Early scientists recognised the importance of *Trifolium* species to farming which aided in the rapid expansion of agricultural production (Caradus et al., 1989). Large investment into the breeding of *Trifolium*, and the resulting volume of research, has put the *Trifolium* genus in the position of the most important pastoral legumes to New Zealand pastures.

### White Clover

White clover improvement was initiated in New Zealand in the 1870s. The breeding methods used in the past for *Trifolium* spp. have produced cultivars that perform in a broad range of climates and farming systems (Caradus et al., 1996a). The breeding techniques prevailing in the 1960s were based on increasing the performance of ecotypes and simple phenotypic selection. Recurrent selection, introduced in the mid-1960s (Williams, 1987), is a method of population improvement through the cyclical selection of the best performing plants within and among families, generation after generation, until the population has the selected desired traits (Hallauer, 1992). Hoyos-Villegas et al. (2018) compared among half-sib (AHS) family selection and among and within half-sib (AWHS) family selection strategies in white clover. AWHS family selection was a superior strategy, especially in early selection cycles. In the first selection cycle, AWHS had 2.5% genetic gain compared to 1% for AHS. Hoyos-Villegas et al. (2019) suggested that recurrent selection increased the rate of genetic gain of yield and persistence in white clover cultivars.

Phenotypic breeding methods have been the most common for population improvement and cultivar development in *Trifolium* breeding. However, the literature is conflicted about how successful these methods have been in increasing the rate of genetic gain. Woodfield and Caradus (1994) stated that the rate of genetic gain for white clover yield and percentage clover in the sward was 6% per decade. However, it was suggested later that the rate of genetic gain was 1.49% per year (Woodfield, 1999). More recently, Hoyos-Villegas et al. (2019) argued that the genetic gain of dry matter yield and clover content of white clover did not reach above 2% per decade. Similar to white clover, the rate of genetic gain in red clover spans a moderate range. The annual rate of genetic gain for forage yield in red clover is estimated at 0.21–1.39% (Riday, 2010; Tucak et al., 2013).

The application of molecular techniques in forage breeding has increased over the last decade (Barker and Warnke, 2001; Woodfield and Brummer, 2001; Wang and Ge, 2006; Zhang et al., 2006; Ravagnani et al., 2012; Walter et al., 2012). The discovery of quantitative trait loci (QTL) for seed and vegetative properties and the development of linkage maps are the first step in the development of marker-assisted selection (MAS) in *Trifolium* breeding programmes (Barrett et al., 2005, 2009; Williams et al., 2007; Faville et al., 2012). The most recent type of molecular markers used in forages is single nucleotide polymorphisms (SNPs). SNPs provide an efficient way of detecting genetic variation. The markers can identify QTL that control traits and if an association is found, are often deployed into MAS breeding programmes (Barrett et al., 2009; Riday, 2011; Biazzi et al., 2017; Knorst et al., 2019). The development of more cost-effective genotyping techniques such as genotyping-by-sequencing (GBS) is thought to increase the uptake of SNP markers. It will also simplify some of the difficulties that occur in deploying markers in outcrossing species (Elshire et al., 2011; Brummer, 2013).

Caradus et al. (1989) reviewed the advances of white clover breeding in New Zealand. The 1920s saw a continual supply of phosphate to New Zealand, allowing increased pasture production and stocking rate (Caradus et al., 1989). The 1930s focussed on enhancing productivity and persistence in existing cultivars (Woodfield and Caradus, 1994). The breeding objectives changed over time with environmental pressure. The 1950s shifted attention to primary physiological and morphological responses to environmental changes. Stolon density and growth of the stolons concerning the production and persistence of white clover became a focal point in the 1970s. In comparison, the 1980s had an emphasis on whole plant studies and the regeneration rates of white clover. In the 1990’s, different farm and pasture management practices, such as utilising cultivars with a specific leaf size, were incorporated into breeding programmes. The levels of cyanogenesis have been incorporated into the aforementioned breeding targets (Caradus et al., 1989).

The leaf size of white clover, large-, medium- or small-leaved, generally determines the type of production system where the cultivar will be utilised. Large-leaved clovers grows tall and upright and have thick stolons and robust roots. They are used frequently in dairy systems as they perform well in rotationally grazed pastures. Although very productive, they have fewer stolons, reducing the persistence in mixed sward. Medium-leaved clovers are the most robust type of clover, performing very well under a range of grazing management systems, except under very close and continuous grazing (Williams, 1987). Often, large- and medium-leaved cultivars are used together in pasture mixes and grazed on dairy pastures. Small-leaved clovers are low-growing with high numbers of leaves and thin, multi-branched stolons. Their compact and low-growing morphology makes it difficult for grazing animals to uproot the plants. Therefore, small-leaved clover have a high tolerance for rigorous defoliation and are often used in sheep grazing systems (Rattray, 2005).

Four main types of white clover were initially classified in New Zealand pastures as below (Caradus et al., 1989):

• Type 1 or “New Zealand Wild White No. 1,” a very productive, cyanogenic, medium-leaved perennial found in fertile old pastures;
• Type 2 or “New Zealand Wild White No. 2,” a perennial, with denser and smaller leaves and therefore less productivity than Type 1.
• Type 3 or “Ordinary New Zealand White,” a non-persistent clover with medium-sized leaves and moderate growth in the first year but poor growth after that.
• Type 4 or “Lax early-flowering New Zealand and ordinary European,” a non-persistent, near-annual type, with small leaves and low productivity.

Type 1 was superior and led the way to certification and commercial production in 1930 (Caradus et al., 1996b). A breeding programme was created to breed the commercialised Type 1 with pedigree New Zealand Certified Mother Seed. The populations were improved continuously until 1957 when the final selection was completed. In 1964 the lines were released as “Grasslands Huia” (Caradus et al., 1989) and the cultivar is still in use today.

Together with improved farm management practices, white clover cultivars with improved persistence have been developed (Rhodes and Harris, 1979; Charlton et al., 1989; Lee et al., 1993; Caradus and Chapman, 1996). Since the release of “Grasslands Huia,” many white clover cultivars have been bred and evaluated. However, the breeding system of white clover presents some unique challenges to breeders. The allotetraploidy in the species arose out of the hybridisation of two diploid ancestors. *T. pallescens* and *T. occidentale* (Williams et al., 2012). This polyploidy and outcrossing nature of white clover has advantages and disadvantages (Comai, 2005). Outcrossing has resulted in high variability both within and between populations, allowing adaptation to a wide range of environments (Hoglund et al., 1985). We showed this high variation through low inbreeding and relatedness level within the collection held at the Margot Forde Germplasm Centre (MFGC) via pedigree analysis (Egan et al., 2019a). In white clover, there is a negative correlation between leaf size and stolon density (Caradus et al., 1996a). Leaf size is used as a measurement of yield under optimal conditions, and stolon density is used as a measure of persistence. Attempts have been made to overcome this issue by improving persistence and herbage yield through crossing small-leaved New Zealand ecotypes and large-leaved introduced germplasm into new populations. This strategy has been successful and has resulted in the development of cultivars such as “Grasslands Demand” and “Grasslands Sustain” (Widdup et al., 1989; Caradus et al., 1997). A study by van den Bosch et al. (1993) showed that selection for elite characteristics of root morphology resulted in a decrease of yield and persistence. Breeders have observed that after the first year, introduced material displays poorer productivity and persistence compared to the New Zealand-adapted cultivars (Caradus et al., 1989).

### Red Clover

The first red clover plants imported into New Zealand were from commercial seed companies in England. There has been a lack of recording of New Zealand ecotypes that are adapted from the imported material (Egan et al., 2019b). The most popular cultivar of choice for New Zealand farmers, originally, was the “Broad Red” type. However, second year red clover populations were weak and sparse. The Montgomery type was slower to establish than Broad Red but provided a much more persistent population (Corkill, 1949; Smith et al., 1985; Woodfield, 1999; Wratt and Smith, 2015). The 1920s saw the beginning of trials to determine the best type of red clover to use for New Zealand pastures, and the differences between Broad Red and Montgomery were apparent. Montgomery proved to be more successful for New Zealand pastures and developed into a breeding programme in the 1930s. In 1937, the seed was certified and was classed as New Zealand Montgomery red clover. It was renamed as “Grasslands Turoa” in 1964 (Wratt and Smith, 2015).

The 1930s showed a good establishment of Broad Red clover and several breeding programmes were developed to increase density and yield (Corkill, 1949; Wratt and Smith, 2015; Egan et al., 2019b). In 1946, the successful parents from these trials were certified to be named as the cultivar “New Zealand Broad Red” clover (Wratt and Smith, 2015). This was later renamed “Grasslands Hamua” in 1964 (Levy, 1970). In European breeding activities, increased persistence has been achieved (Abberton et al., 2011; Marshall et al., 2017).

In New Zealand, the development of tetraploid red clover began in 1954, and by 1972 “Grasslands Pawera” was commercialised. Phenotypically, this cultivar is described as a mix between “Grasslands Turoa” and “Grasslands Hamua” (Wratt and Smith, 2015). “Grasslands G27” is another example of tetraploid (Rumball et al., 1997; Wratt and Smith, 2015). “Grasslands G27” was bred from “Grasslands Pawera” with the breeding target of reduced formononetin. Formononetin is the causative hormone that is related to reduced conception and ovulation rates in ewes. Selection cycles continued for seven generations and the final elite population had less than half of the level of formononetin than “Grasslands Pawera” under grazing sward conditions (Rumball et al., 1997).

Tetraploids (2*n* = 28) have distinct advantages when compared to diploids (Taylor and Quesenberry, 1996b). First, the doubling of chromosomes interrupts the alleles that control self-fertility and often tetraploids exhibit increased numbers of self-fertile plants compared to diploids (Drach et al., 1986). Second, the tetraploid red clover cultivars often outperform the diploid cultivars in dry matter yield and disease resistance (Joensson, 1985; Arseniuk, 1989; Yamada and Hasegawa, 1990; Taylor and Quesenberry, 1996a; Liatukas and Bukauskaitë, 2012). Third, tetraploids are usually larger in most plant structures, including flowers and seeds (Nikovitz, 1985). However, tetraploids have lower seed production compared to diploids, so higher sowing rates are needed. The lowered seed yield of tetraploids has been a major limiting factor in the production of these cultivars (Taylor and Quesenberry, 1996a).

Red clover improvements have been focussed on yield and persistence (Smith et al., 1985; Taylor and Quesenberry, 1996a; Ford and Barrett, 2011; Marshall et al., 2017). The need for increased persistence was recognised in the 1930s and similar to white clover, breeding efforts began by focussing primarily on plant morphology. However, breeding for persistence in red clover has proved challenging. Cultivar development has progressed by selecting for targetted traits such as increased “creeping” phenotypes and stoloniferous features to increase persistence (Williams et al., 2007). “Grasslands^TM^ Relish” is the most recent New Zealand red clover cultivar in the market. Relish can persist in pastoral systems for 3–4 years; a significant increase than other red clover cultivars (Ford and Barrett, 2011).

Red clover breeding has been more prominent internationally than in New Zealand and is reflected in the abundance of the plant distribution globally (McKenna et al., 2018). Many of the earlier developed cultivars are early-flowering types and lack persistence, as they are most commonly used as a forage supplement crop rather than in a long-term grazing system (Taylor and Quesenberry, 1996a). Despite its lack of persistence, red clover is considered an important component in pastoral systems throughout New Zealand. The cultivars released from New Zealand are agronomically similar to overseas cultivars but are adapted to New Zealand conditions (Egan et al., 2019b). All of the red clover cultivars in New Zealand are synthetics and have been created through the open pollination of multiple elite parents (Wratt and Smith, 2015).

The most prominent finding in red clover has been the discovery of phytoestrogens (Taylor and Quesenberry, 1996a). Phytoestrogens are a plant-derived oestrogen that is structurally and functionally similar to mammalian oestrogens (Patisaul and Jefferson, 2010). Many phytoestrogens are phenolic compounds that belong to the isoflavones group, which is present in many legume species. Further research showed a strong link between high levels of phytoestrogens, specifically formononetin, in red clover, and decreased efficiency in ewe fertility (Rumball et al., 1997). Grazing management has allowed better control over the exposure to formononetin (Shackell et al., 1993a,b). However, the more recent red clover cultivars have been bred with lower levels of phytoestrogen. Five breeding cycles are usually needed to reduce the formononetin to a safe level for the sheep (Wratt and Smith, 2015).

### Minor *Trifolium* Species

The under-utilisation of other *Trifolium* species in New Zealand has encouraged research into their potential use in pastoral systems. *T. subterraneum* and *T. ambiguum* are two clover species that are utilised widely in some countries, showing the potential to introduce the species into farming systems in New Zealand (Abberton, 2007; Nichols et al., 2014a). However, the utilisation of minor *Trifolium* species in New Zealand is still very low.

The majority of *T. subterraneum* breeding has been implemented in Australia by improving local germplasm (Taylor, 1985). Francis et al. (1970) summarised the key breeding targets for *T. subterraneum* as low oestrogen content, leaf marks linked to physiological traits, maturity, burr burial, physiological seed dormancy, hard seededness and resistance to diseases and insects. Similar to red clover, *T. subterraneum* has high levels of formononetin which is generally selected against early in the breeding programme (Nicholas et al., 1981). Trials across New Zealand have been evaluated to select *T. subterraneum* lines that have improved persistence and dry matter production (Dodd et al., 1995a,b, c). Recent studies have identified Australian cultivars that are best adapted to New Zealand dryland pastoral systems (Lucas et al., 2015; Olykan et al., 2018). However, more improvement is needed for developing cultivars that fit better to New Zealand environments.

Another important breeding objective in *T. subterraneum* breeding programmes has been resistance to pathogens and diseases. Pathogens such as clover scorch (*Kabatiella caulivora*), rust (*Uromyces trifolii-repentis*), powdery mildew (*Oidium* sp.), and root rot (*Fusarium avenaceum* and *Pythium irregulare*) are common in subterranean clover. There have been many success stories in developing resistant lines (You et al., 2005a,b; Nichols et al., 2014a).

*Trifolium subterraneum* was the first annual *Trifolium* species to have a draft genome sequenced. As it is an annual, diploid (2*n* = 16) species with a small genome (540 Mbp), it has been an attractive species to use as a model *Trifolium* (Hirakawa et al., 2016). Using *T. subterraneum* as a model to understand the genetics of traits of interest will provide a pathway to understanding traits in the more genetically complex species from the genus *Trifolium* and tribe *Trifoliae*.

*Trifolium ambiguum* has the potential to become a major forage legume in New Zealand (Taylor and Smith, 1997; Watson et al., 1997; Nguyen et al., 2020). *T. ambiguum* exists in diploid, tetraploid, and hexaploid forms and is highly self-incompatible at all ploidy levels. Although incompatibility exists between ploidy levels, there have been interploidal hybrids produced (Kannenberg and Elliott, 1962). The significant strengths of *T. ambiguum* are its longevity and persistence under intensely grazed pastoral systems (Spencer et al., 1975; Dear and Zorin, 1985; Sheaffer and Marten, 1991; Sheaffer et al., 1992; Virgona and Dear, 1996). The breeding efforts thus far have focussed on major agronomic traits such as seed and forage yield, and early and late flowering time, as well as the more complex traits of drought resistance and compatibility with *Rhizobium* strains (Taylor and Smith, 1997; Williams et al., 2007; Nguyen et al., 2020).

*Trifolium ambiguum* has been a considerable source of new variation generated through hybridisation with white clover (Meredith et al., 1995; Williams et al., 2019). One of the major goals of *T. repens* × *T. ambiguum* hybrids is to introgress the large root system of *T. ambiguum* while keeping the agronomic performance of white clover (Abberton et al., 1998, 2002; Widdup et al., 2003; Williams and Hussain, 2008; Williams et al., 2019). The biggest challenges in these hybrids are maintaining seed production, slow establishment and producing viable hybrids (Meredith et al., 1995; Taylor and Smith, 1997). *T. ambiguum* could also be a potential source of virus resistance to white clover. Barnett and Gibson (1975) reported that *T. ambiguum* showed resistance to a range of viruses including alfalfa mosaic, yellow bean mosaic, peanut stunt and white clover mosaic viruses. Although *T. ambiguum*, both by itself and as a hybrid, shows promise to become a productive forage legume in New Zealand pastoral systems, white and red clover remain very popular in the farming community due to their continuing high performance (Taylor, 2008).

The breeding system of *T. arvense* is both selfing and outcrossing (Palmer, 1972). The breeding and research into *T. arvense* are limited. However, a study by Hancock et al. (2012) used genetic modification (GM) to integrate the transcription factor, TaMYB14, from *T. arvense* into *T. repens*. TaMYB14 is involved in the regulation of proanthocyanidin (PA) biosynthesis in legumes. PAs are polyphenolic secondary metabolites in plants and are associated with providing defence against pathogens and herbivores (de Colmenares et al., 1998; Aziz et al., 2005; Dixon et al., 2005). The GM clover has shown promising results in decreasing methane emissions and reducing bloat in livestock (Hancock et al., 2012).

*Trifolium hybridum* is a self-incompatible, highly outcrossing species. Cultivars are either diploid (2*n* = 16) or tetraploid (2*n* = 36). There have been limited breeding programmes with *T. hybridum*, but wide variability has been shown in agronomic traits, except for persistence (Matthews and Battle, 1951; Townsend, 1964; Widdup and Ryan, 1994; Petroviæ et al., 2014). However, inbreeding in *T. hybridum* reduces the persistence (Matthews and Battle, 1951). Townsend and Remmenga (1968) assessed both selfed and outcrossed populations to measure the effect that selection for persistence had on the outcrossed progeny. The outcrossed populations had more persistence, but the gain was not enough to continue with selections.

*Trifolium medium* is highly self-incompatible and has 2*n* chromosome numbers ranging from 64 to 80 (Quesenberry and Taylor, 1977). *T. medium* has been involved in several breeding programmes (Taylor et al., 1984). A draft genome of *T. medium* has been assembled to accelerate breeding advancements in clover breeding (Dluhošová et al., 2018). There have been attempts to produce *T. medium* × *T. repens* and *T. medium* × *T. pratense* hybrids, but they have been unsuccessful (Anderson and Taylor, 1974; Kazimierska, 1978). *T. medium* has been successfully crossed with *T. sarosiense* to bridge the genetic gap between *T. medium* and *T. pratense* (Quesenberry and Taylor, 1977). The breeding target of *T. pratense* × *T. medium* hybrids is to incorporate increased perenniality into *T. pratense* from *T. medium* (Abberton, 2007).

The main breeding programme for *T. hybridum* in New Zealand was for the development of G41 zigzag clover. This programme focussed more on seed-setting than agronomic vigour (Rumball and Claydon, 2005). For *T. medium* to be used as a potential forage legume in New Zealand pastoral systems, more research into seed traits and the agronomic and management practices in high-country systems is needed (Daly and Mason, 1987; Taylor, 2008).

## Role of Genebanks and Germplasm Prospecting in *Trifolium* Improvement

Modern agricultural and agronomic practices have become very intensive, exploiting almost all areas of farming. Germplasm centres worldwide will play a vital role in food security for the future (National Research Council (US) Committee on Managing Global Genetic Resources: Agricultural Imperatives, 1991; Sharma, 2017; Díez et al., 2018). Approximately 7.4 million germplasm accessions from different plant species have been collected worldwide (FAO, 2010). During the 1970s, there was a rapid increase of the establishment of germplasm centres in developing countries, and today a large volume of germplasm is held in these centres (Plucknett et al., 1983; Nass et al., 1993; Morris and Greene, 2001; Rodriguez-Medina et al., 2019). The role of germplasm in the improvement of plant cultivars has been widely recognised, however, without thorough analysis, the germplasm contained in the centres are of limited use.

Forage legumes are important components of agrobiodiversity, especially in countries where livestock production contributes largely to their GDP. A report produced by FAO (2010) stated that global germplasm holdings had 651,024 forage accessions (accessions are defined as a collection of seed from a defined lineage or cross); 35% were wild species, 13% were landraces (landraces are locally adapted populations of species), 4% were advanced cultivars, 3% were breeding materials, and 45% were others. Of the total genebank accessions collected over the period of 1996–2007, 15% were forages and, in total, contribute to 9% of the major crop groups in total *ex situ* collections. According to the latest available data, New Zealand holds the largest collections of temperate forage species (Global Crop Diversity Trust, 2020).

The MFGC is the national forage germplasm bank in New Zealand and holds a large *Trifolium* collection (Table 1; Egan et al., 2019a,b). A key example of the success of germplasm utilisation from the MFGC is the development of the red clover cultivar, “Grasslands^TM^ Relish” (Ford and Barrett, 2011). Relish was developed on the basis of the initial screening of wild germplasm at the MFGC. However, overall, there is a low utilisation of germplasm in New Zealand *Trifolium* breeding programmes (Egan et al., 2019a,b, 2020).

**TABLE 1:** The breakdown of the number of *Trifolium* accessions recorded at the Margot Forde Germplasm Centre.

**Genus**	**Total**	**Breeding/Research**	**Commercial**	**Collected**	**Exchange**
*Trifolium repens*	30,047	24,696	1,522	2,442	1,387
*Trifolium pratense*	6,953	4,386	1,069	802	696
*Trifolium subterraneum*	1,520	412	243	235	630
*Trifolium ambiguum*	1,162	917	37	141	67
*Trifolium hybridum*	582	309	43	153	77
*Trifolium dubium*	252	180	0	32	40
*Trifolium medium*	247	107	12	80	48
*Trifolium arvense*	220	18	2	140	60
*Trifolium occidentale*	3,215	2,997	0	184	34
*Trifolium repens* × *occidentale*	2,833	2,833	0	0	0
Other *Trifolium* interspecific hybrids	4,252	4,245	5	0	2
Other *Trifolium* species (233 species)	4,451	171	158	1,701	2,421

*Breeding/Research accessions includes some cultivars and exchange germplasm and are not publicly available or viewable. Commercial lines are cultivars that were commercially released. Collected accessions are accessions that were collected on overseas trips. Exchange accessions are germplasm from other sources, including other international institutions.*

### Core Collections

Core collections effectively makes collected germplasm usable and reduces the scale of management required in a genebank and are often used in diversity studies for traits to assess if there is suitable variation for plant breeding Frankel and Brown (1984) proposed the development of core collections as a subset containing a minimum set of accessions that represent maximum diversity of the original collection. The core collection concept has gained more momentum in the last two decades (Basigalup et al., 1995; Johnson and Hodgkin, 1999; Johnson et al., 1999; Marita et al., 2000; Ghamkhar et al., 2008). It was originally used as a management technique (Allard et al., 1993). However, it is increasingly being used for association mapping, trait-gene links for targetting material back in to the original collection and gap finding in the original collection (Abdurakhmonov and Abdukarimov, 2008; Neumann et al., 2011; Kloth et al., 2012; Riedelsheimer et al., 2012; Gupta et al., 2019). The highly characterised accessions within the collection can be used to inform decisions in breeding programmes (Zhang et al., 2019; Abdi et al., 2020).

In New Zealand, the development of a white clover core collection is at an advanced stage. However, there are no immediate plans for a red clover core collection. The core collections will increase the level of germplasm utilisation in the MFGC, and natural variation within the primary gene pool of *Trifolium* species will be exploited. There are some concerns about core collections potentially leading to the negligence of the original collection. A core collection that does not encompass a considerable amount of the whole collections’ diversity would not serve a purpose (Brown, 1989a). To overcome the potential loss of diversity, Brown (1989b) suggested a simple random sampling method which had high retention of diversity statistics. To ensure that core collections are not formed using misleading information, deep characterisation of the germplasm data associated with the accessions compiled in the core collection is needed (Allard et al., 1993; Singh et al., 2019).

### Characterisation of Germplasm in Genebanks

Most of the genetic variation present in genebanks are absent in breeding programmes but could have useful variation for future breeding programmes (Dwivedi et al., 2017; Egan et al., 2019a,b). Although there is increased genetic and phenotypic data on traits, knowledge of all of the accessions in any genebank as a whole, is lacking (Bretting, 2018). When an accession is collected, it is linked with passport data (latitude, longitude, and geographical information), and ecological data. Each time the accession is grown in a trial or for regeneration, it is critical that more phenotypic data is collected to characterise the accession as much as possible in a genebank (Allard et al., 1991). This information may eventually be of use to agricultural needs and breeding programmes (Ghamkhar et al., 2015; Bretting, 2018).

While New Zealand invests heavily into *Trifolium* research, several other countries have also characterised *Trifolium* germplasm collections. Zhang et al. (2010) conducted the first study of determining genetic variation of Chinese local white clover germplasm. A study from India by Gupta et al. (2017) assessed genetic diversity of a global red clover collection, procured from the USDA, using SSR markers. Both studies revealed that the accessions were diverse in both morphological traits and molecular marker patterns. A collaborative study between Spain, Colombia and the United States applied predictive characterisation based on ecogeographic information to evaluate target traits in forage genetic resources (Sánchez et al., 2019). Such predictive modelling is a low-cost option to increase germplasm characterisation in relation to a specific trait. More recently, Jones et al. (2020) described a thorough characterisation of European and Asian red clover germplasm through molecular techniques.

## Past Successes of Using Germplasm in Forage Breeding

Utilising germplasm held in genebanks has been crucial for the improvement of plant species, and this has been recognised for many years (Ghimiray and Vernooy, 2017). Hybridising species with germplasm or wild relatives has been successful in many plant species. Broadening the breeding pool has introduced increased resistance, yield and variation to be incorporated into populations. Genetic variation is crucial to have in a population, as without variation, the genetic gain cannot be realised (Williams, 1987). Forages have been a group of interest for hybridisation with wild relatives due to the slow rate of genetic gain from conventional breeding (Nass et al., 2012; Hoyos-Villegas et al., 2019). Woodfield and Brummer (2001) suggests that the limited rate of genetic gain could be increased by changing from synthetic varieties to hybrid varieties and could provide better control over traits that would improve performance in the species.

The utilisation of species that occur in the primary and secondary gene pool to the target species have been useful to generate new variation to widen the genetic base and improve species. The primary gene pool is characterised by accessions from the same species, whilst the secondary gene pool are different species than the target species but can still produce fertile hybrids (Acquaah, 2012). Ellison et al. (2006) developed the “white clover species complex,” outlining the species that are closely related and can cross with another in the complex. The key example of using germplasm in *Trifolium* is the interspecific hybrids of *T. repens* with *T. ambiguum, T. uniflorum*, and *T. occidentale* (Marshall et al., 2008, 2015; Williams and Hussain, 2008; Widdup and Barrett, 2011; Williams et al., 2013; Nichols et al., 2014b,c,d,e, 2015; Williams, 2014; Hussain et al., 2016; Lloyd et al., 2017).

The ploidy of the related species is challenging when developing hybrids. *T. uniflorum* is a tetraploid, *T. occidentale* is a diploid clover, and *T. ambiguum* can exist in the ploidy forms of 2*x*, 4*x*, and 6*x* (Williams et al., 2006; Abberton, 2007; Jahufer M.Z.Z. et al., 2013). The 6*x* form of *T. ambiguum* is best suited for agronomic conditions, but this has not been successfully crossed to white clover to produce fertile hybrids. Williams and Hussain (2008) overcame the genetic bridge of *T. ambiguum* × *T. repens* by doubling the chromosome number and then backcrossing to white clover until stable tetraploid hybrids were produced. More recently, a study by Williams et al. (2019) used 2*x T. occidentale* × 6*x T. ambiguum* as a genetic bridge to hybridise the two species and produce one gene pool.

The advancements of the interspecific hybrids have been successful. Nichols et al. (2014c) showed that *T. repens* × *T. uniflorum* BC1 hybrids outperform white clover in drought conditions. Nichols et al. (2014b) identified some *T. repens* × *T. uniflorum* interspecific hybrids that were more tolerant of low external phosphate supply. Nichols et al. (2015) studied the response of *T. repens* × *T. uniflorum* BC_1_ and BC_2_ interspecific hybrids and white clover cultivars in response to drought conditions. The BC_1_ hybrids maintained photosynthesis under drought and had the highest levels of biochemical compounds that enable a plant to perform in water-stressed environments. The next steps for the progression of the interspecific hybrids are the continuation of selection cycles to improve populations in agronomic traits.

Attempts at hybridisation between other *Trifolium* species have seen some success (Ferguson et al., 1990). *T. nigrescens* has demonstrated a close affinity with *T. repens* and has had successful crosses (Brewbaker and Keim, 1953; Hovin, 1962). *T. nigrescens* has several useful reproductive traits that could benefit *T. repens*, including a prolific number of inflorescences. Marshall et al. (1995) showed that *T. repens* × *T. nigrescens* hybrid progeny showed intermediate reproductive phenotypes and was a significant increase from *T. repens*. More recently, Marshall et al. (2008) showed that introgression of reproductive traits from *T. nigrescens* to *T. repens* increased the seed yield. Malaviya et al. (2018) investigated the interspecies incompatibility and affinity between *T. alexandrinum* and 22 *Trifolium* species. Although there was incompatibility among most of the crosses, embryo rescue and intensive crossing produced successful hybrids.

## Plant Breeding Avenues for Germplasm Exploration and Retaining and Maximising Diversity

Plant breeding relies on diversity in populations to succeed. Without heritable phenotypic variation in the populations, there will be little or no increase in population performance (Govindaraj et al., 2015). However, diversity is a term that has no clear definition. It is broadly referred to as any variation at any phenotypic or molecular level in the species at any given time (Fu, 2015).

Crossing elite germplasm lines to increase the performance of populations has produced cultivars across all major species. However, with the increased loss of genetic diversity in cultivars (Van de Wouw et al., 2010; Keneni et al., 2012), there is an urgent need to introduce wild relatives to widen the genetic base of populations. The hybridisation of wild germplasm into adapted germplasm is generally performed through backcrossing to the elite parent or by recurrent selection. Williams (2010) reviewed the potential value of related species to important crop plants. The related species to the plant of interest comprise the secondary and tertiary gene pools. The hybridisation of a crop with a related species, often termed “crop-wild hybridisation,” to enhance the production has been well practiced (Hoc et al., 2006; D’Andrea et al., 2008; Uwimana et al., 2012). Wild relatives can be a large resource for useful genes due to the adaptation of these plants to varying conditions in their native habitat, and such as temperature, salinity, pH, and geography. Although the amount is unknown, it is clear that the wild relatives hold large economic potential (Feldman and Sears, 1981).

The development of interspecific hybrids through pre-breeding has many challenges, including infertility, linkage drag, inbreeding, and crossing incompatibility (Acquaah, 2012). Despite these challenges, pre-breeding, the early activities used to characterise germplasm that identifies useful characteristics (Acquaah, 2012), has become frequently utilised to develop populations with increased variation (Nass and Paterniani, 2000; Acosta-Gallegos et al., 2007; Sharma et al., 2013). Identifying genetic variation and utilising information from genebanks to plant breeding programmes is an important strategy for continuing crop genetic improvement (Sehgal et al., 2015).

The two major *Trifolium* species, red and white clover, are recently domesticated forage crops, unlike most other crops. This suggests that they are similar to related wild species (Harlan, 1983). Morris and Greene (2001) defined the components and sizes of the subpools in red and white clover. Cultivars and selection-based populations comprise the first and second components and are larger than the third component of landraces. The fourth and fifth components containing naturalised populations would be large and have the largest amount of diversity. The first, second and third components have been suggested to produce the largest number of cultivars (Rumbaugh, 1990, 1991). Despite the wide variation available in red and white clover, Egan et al. (2019a) showed the clustering among accessions in the MFGC and how one cluster has been utilised to produce the majority of white clover cultivars.

### Pedigree Maps and Analysis

Pedigree analysis has been popular in breeding programmes to monitor crosses between individuals to maximise diversity. In the literature, there are more reports of pedigree analysis in animals (Roughsedge et al., 1999; Valera et al., 2005; Leroy et al., 2006; Calboli et al., 2008; Cervantes et al., 2008; Hamann and Distl, 2008; Graczyk et al., 2015) than in crops (Souza and Sorrells, 1989; Gizlice et al., 1994; Sneller, 1994; Navabi et al., 2014). Although pedigree analysis is typical in animals as both parents are known, it is an important method that enables the tracking of crosses of plant germplasm accessions (Philipp et al., 2018).

The development of a pedigree map allows a visual representation of population structure and determines the effect of human-based decision making. Pedigree maps show past breeding performed, and breeding pools can be identified (Holland et al., 2003). It is not crucial that all of the mating relationships are recorded, although indeed helpful (Shaw et al., 2014). Phenotypic and genotypic information can be added to pedigrees to add depth and knowledge which aids in selection cycle decision making (Breseghello and Coelho, 2013), and can increase the genetic gain in a breeding cycle (Riday, 2011). However, the large and complex nature of pedigree data sets provides perceptive limitations in building, visualising and analysing large pedigrees (Shaw et al., 2014). Genetic factors of populations can be described by deriving pedigree-related coefficients such as kinship, inbreeding, the effective number of founders and the effective population size (Falconer, 1996; Bernardo, 2010; Voorrips et al., 2012). The information obtained from pedigree maps and analysis enables faster and more efficient breeding decisions (Holland et al., 2003). Pedigree analysis on white clover (Egan et al., 2019a), red clover (Egan et al., 2019b), and minor *Trifolium* species (Egan et al., 2020) germplasm collections in New Zealand have been performed. High diversity was found within all collections.

Where available, molecular tools are utilised with pedigrees in mixed models (Graner et al., 1994; Melchinger et al., 1994; Bink et al., 2002; Janick, 2003; Dreisigacker et al., 2004). The main limitation of pedigree analysis based only on parental data is the accuracy of the parental data (Egan et al., 2019a). The integration of molecular markers to validate pedigrees has become increasingly popular to confirm results (VandeBerg et al., 1990; Smith et al., 2000; Paiva et al., 2011; Daetwyler et al., 2012).

### Genome-Wide Association Study

The ability to describe the relationship between genotype and phenotype has become increasingly important with increased pressure on the performance of crops (Liu et al., 2018). A genome-wide association study (GWAS) is a method that involves scanning the genome using DNA markers to detect associations with phenotypic traits. GWAS was first developed in human genetic before becoming utilised in plant species. It is a powerful tool to identify QTL and causative SNPs in both simple and complex traits.

GWAS has become common for analysing simple traits and for furthering the understanding of the genetic architecture of complex traits, i.e., the number of loci that contribute to a trait and the relative contribution to the phenotype. Complex traits are controlled by many rare variants having a sizeable phenotypic effect or, common variants resulting in a small phenotypic effect (Korte and Farlow, 2013). Many of the traits that are breeding targets in forages are complex, so GWAS is promising to identify genomic regions controlling traits (Korte and Farlow, 2013). However, simple traits underpinned by a small number of loci with large effect sizes, are typically best suited for GWAS. The effect of rare variants is difficult to prove through GWAS due to lack of statistical power (Acquaah, 2012).

Genotyping-by-sequencing has become a standard sequencing method to use in GWAS studies due to the low cost, high throughput and robustness of the method (Sonah et al., 2015; Sakiroglu and Brummer, 2017; Han et al., 2018). Restriction enzymes are used to reduce genome complexity and the number of repetitive elements. GBS was first developed by Elshire et al. (2011) and is suitable for outbreeding populations as genome-wide allele frequency profiles can be calculated in pooled samples. However, GBS can pose challenges in the form of low sequencing depth and missing genotype calls (Ashraf et al., 2016).

The history and successful development of GWAS techniques have been well documented (Ikegawa, 2012; Visscher et al., 2017). GWAS have been successful in identifying novel variant-trait associations (Tam et al., 2019; Jones et al., 2020) and allowed marker-assisted selection breeding programmes in forages to be developed (Hayward et al., 1994; Barrett et al., 2001, 2006, 2009; Dolstra et al., 2007; Roldán-Ruiz and Kölliker, 2010; Riday, 2011). However, the number of GWAS studies in forages compared to other crops is low. The highly heterozygous and outcrossing nature of forages makes finding and validating associations more complex compared to other crops that have a closed mating system. Although the overall number of studies is low, there have been numerous GWAS studies in alfalfa that have identified regions of the genome that control forage yield, nutritive value (Sakiroglu et al., 2012), forage quality traits (Biazzi et al., 2017), as well as plant growth and forage production under abiotic stresses (Liu and Yu, 2017). Significant marker-trait associations have been identified in ryegrass (Brazauskas et al., 2011; Fè et al., 2015; Arojju et al., 2016). There has only been one reported GWAS in white clover where Inostroza et al. (2018) identified 53 loci associated with cold-tolerance traits.

## Future Directions

Prospects for tools and breeding targets for future *Trifolium* improvement could include landscape genomics, disease and pest resistance, male sterility, improvement of the phenomic and genomic tools available to breeders, adaptation to climate change, methane output traits, and isoflavones.

Landscape genomics, the study of adaptive evolution in species in response to the landscape, has increased in uptake in the past decade (Li et al., 2017). The ability to link the genotype to the phenotype, or ecosystem service, will allow a better measurement of the holistic behaviour of the species and the maximisation of ecological services. Incorporating landscape genomics into pre-breeding programmes will provide a more informed view of *Trifolium* breeding and further characterise germplasm.

While disease and pest resistance have historically been at the forefront of *Trifolium* breeding programmes (Wong and Latch, 1971; Voisey et al., 1994, 2001; Murray, 1996; Woodfield and Caradus, 1996; Eerens et al., 2001; Scott et al., 2001), in recent decades, production-based traits have become more prominent breeding targets (Annicchiarico, 2003; Barrett et al., 2005, 2009; Cogan et al., 2006). The loss of focus of disease and pest resistance is a weakness to *Trifolium* breeding programmes, as warmer temperatures induced by climate change are predicted to provide ideal conditions for pests and diseases (Rosenzweig et al., 2001; Juroszek and Von Tiedemann, 2011; Luck et al., 2011; Bebber et al., 2013).

The possibility of using new chemical agents for the induction of male sterility could play a major role in hybrid clover seed production in the future (Easterly et al., 2019). Sterile male plants increase the feasibility of producing hybrids of cross-pollinated species that traditionally utilise a population improvement method. As the male aspect is controlled, the improvement of both parents and the need for a population improvement scheme is reduced.

Continuing to improve the phenomic and genomic tools available to breeders will continue to be an area of research that is needed to accelerate the rate of breeding programmes (Varshney and Dubey, 2009; Perez-de-Castro et al., 2012). Continuing to map loci to the genome will further improve the genomic tools available to breeders. To match the tools available to genetic gain, better phenotyping capacity and tools will be crucial in the future. Although there is variation available in germplasm banks, there is currently no high-throughput phenotyping methods for structural traits, i.e., root characteristics, as opposed to morphological traits, and this will be an undesirable bottleneck in improving germplasm. These methods will not only need to focus on carbon uptake but water, nitrogen and phosphorus too. Developing high-throughput phenotyping tools will allow deeper characterisation of *Trifolium* germplasm and could increase germplasm utilisation.

Improved traits related to climate change and sustainability in animal production and health will be a key area of future research. Adaptation to climate change through traits such as drought tolerance and increased nutrient efficiency will be a prominent area of research for *Trifolium* breeding programmes. White clover has a shallow root system (Caradus et al., 1989) and exploring the natural variation in root traits will be key for adaptation to change in hill country farms on the east coast of New Zealand. Currently, both red and white clover have high methane output from the animal gut (Waghorn et al., 2002; Hammond et al., 2011). There is increased urgency to reduce methane emissions from farming (Moumen et al., 2016), so germplasm screening for accessions with less methane emission potential will be critical. A continuing effort will be needed to screen red clover germplasm for low levels of isoflavone content to reduce the risk of ewe infertility (Kelly et al., 1979; Mustonen et al., 2014).

## Conclusion

As the global population increases, so will the intensification of agriculture. Like many major crops around the world, revitalisation and expansion of *Trifolium* breeding will have significant challenges. Breeding targets will encompass increasing pest and disease resistance and adaptation to anthropogenic climate change, while elevating forage quality and production. There are also the impending sustainability goals of countries with a policy to have a minimal environmental footprint in the future.

The history and challenges of *Trifolium* breeding can provide context as to why there has been a slow rate of genetic gain. The development of novel populations, such as *Trifolium* interspecific hybrids with better performance in adverse conditions could reduce farm input. Utilising germplasm will become crucial to accelerate the pace for reaching the global goals of increased productivity and sustainability. Both the public and private sectors will have to work in harmony to meet these goals, utilising these untapped resources while developing innovative methods in forage breeding.

Increasing funding for pre-breeding research could generate more genetic diversity potentially of use in breeding programmes. This may lead to the next generation of clovers adapted to future climate scenarios. Pedigree analysis and GWAS are among methods that can help genebank managers and breeders to understand the variation and population structure present in the germplasm of each species. Such methods could also inform intra- and inter-population recurrent selection progeny-based methodologies.

## Author Contributions

LE wrote the manuscript, provided intellectual input, and proofread the manuscript. RH provided comments and improved the writing. KG provided support and comments. VH-V conceived the idea and provided input and supported the project. All authors contributed to the article and approved the submitted version.

## Conflict of Interest

The authors declare that the research was conducted in the absence of any commercial or financial relationships that could be construed as a potential conflict of interest.

## Abbreviations

AHS: among half-sib
AWHS: among and within half-sib
SNP: single nucleotide polymorphism
MAS: marker-assisted selection
QTL: quantitative trait loci
GBS: genotyping-by-sequencing
GM: genetic modification
MFGC: Margot Forde Germplasm Centre
G × E: genotype × environment
GWAS: genome-wide association study.
